# Supplementary material for: Molecular Characterization of Small Ruminant Lentiviruses Isolated from Polish Goats with Arthritis
Source: Viruses. 2022 Mar 31;14(4):735. doi: 10.3390/v14040735 (PMC9032046; doi:10.3390/v14040735)
Supplement: Supplementary file 1 [file viruses-14-00735-s001.zip › viruses-1634448-supplementary.pdf]

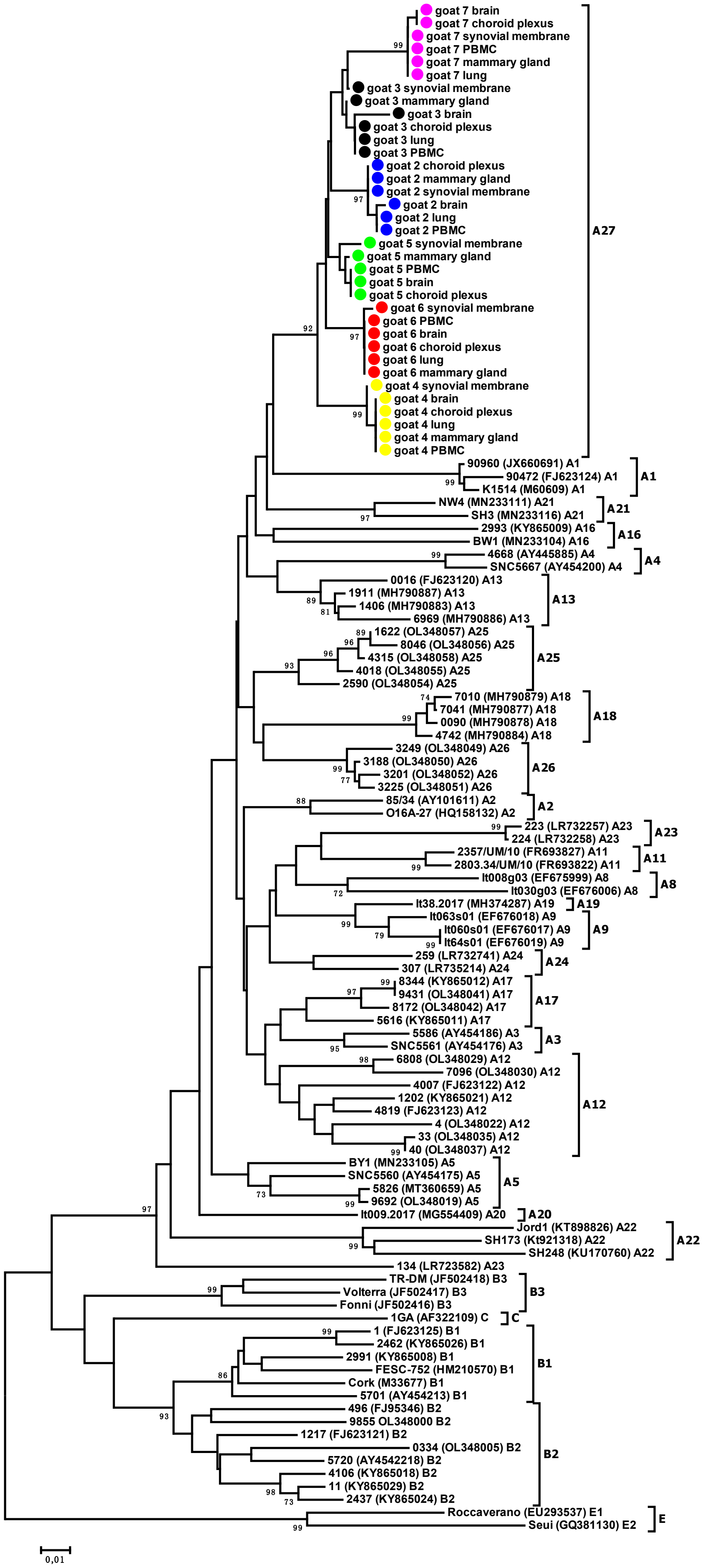
goat 7 brain
goat 7 choroid plexus
goat 7 synovial membrane
goat 7 PBMC
goat 7 mammary gland
goat 7 lung
goat 3 synovial membrane
goat 3 mammary gland
goat 3 brain
goat 3 choroid plexus
goat 3 lung
goat 3 PBMC
goat 2 choroid plexus
goat 2 mammary gland
goat 2 synovial membrane
goat 2 brain
goat 2 lung
goat 2 PBMC
goat 5 synovial membrane
goat 5 mammary gland
goat 5 PBMC
goat 5 brain
goat 5 choroid plexus
goat 6 synovial membrane
goat 6 PBMC
goat 6 brain
goat 6 choroid plexus
goat 6 lung
goat 6 mammary gland
goat 4 synovial membrane
goat 4 brain
goat 4 choroid plexus
goat 4 lung
goat 4 mammary gland
goat 4 PBMC
A27
90960 (JX660691) A1
90472 (FJ623124) A1
K1514 (M60609) A1
A1
NW4 (MN233111) A21
SH3 (MN233116) A21
A21
2993 (KY865009) A16
BW1 (MN233104) A16
A16
4668 (AY445885) A4
SNC5667 (AY454200) A4
A4
0016 (FJ623120) A13
1911 (MH790887) A13
1406 (MH790883) A13
6969 (MH790886) A13
A13
1622 (OL348057) A25
8046 (OL348056) A25
4315 (OL348058) A25
4018 (OL348055) A25
2590 (OL348054) A25
A25
7010 (MH790879) A18
7041 (MH790877) A18
0090 (MH790878) A18
4742 (MH790884) A18
A18
3249 (OL348049) A26
3188 (OL348050) A26
3201 (OL348052) A26
3225 (OL348051) A26
A26
85/34 (AY101611) A2
O16A-27 (HQ158132) A2
A2
223 (LR732257) A23
224 (LR732258) A23
A23
2357/UM/10 (FR693827) A11
2803.34/UM/10 (FR693822) A11
A11
It008g03 (EF675999) A8
It030g03 (EF676006) A8
A8
It38.2017 (MH374287) A19
A19
It063s01 (EF676018) A9
It060s01 (EF676017) A9
It64s01 (EF676019) A9
A9
259 (LR732741) A24
307 (LR735214) A24
A24
8344 (KY865012) A17
9431 (OL348041) A17
8172 (OL348042) A17
5616 (KY865011) A17
A17
5586 (AY454186) A3
SNC5561 (AY454176) A3
A3
6808 (OL348029) A12
7096 (OL348030) A12
4007 (FJ623122) A12
1202 (KY865021) A12
4819 (FJ623123) A12
4 (OL348022) A12
33 (OL348035) A12
40 (OL348037) A12
A12
BY1 (MN233105) A5
SNC5560 (AY454175) A5
5826 (MT360659) A5
9692 (OL348019) A5
A5
It009.2017 (MG554409) A20
A20
Jord1 (KT898826) A22
SH173 (Kt921318) A22
SH248 (KU170760) A22
A22
134 (LR723582) A23
TR-DM (JF502418) B3
Volterra (JF502417) B3
Fonni (JF502416) B3
B3
1GA (AF322109) C
C
1 (FJ623125) B1
2462 (KY865026) B1
2991 (KY865008) B1
FESC-752 (HM210570) B1
Cork (M33677) B1
5701 (AY454213) B1
B1
496 (FJ95346) B2
9855 OL348000 B2
1217 (FJ623121) B2
0334 (OL348005) B2
5720 (AY4542218) B2
4106 (KY865018) B2
11 (KY865029) B2
2437 (KY865024) B2
B2
Roccaverano (EU293537) E1
Seui (GQ381130) E2
E
0,01

Figure S1. Neighbor-joining phylogenetic tree based on alignment of CA fragment of *gag* gene. Sequences from this study are labeled by black circles. Subtypes are marked in different colors. Numbers at the branches indicate the percentage of bootstrap values obtained from 1000 replicates. Bootstrap values >70% are shown. In the present study, the SRLV subtypes found by Colitti et al. [23] were renamed from A18 to A19 and from A19 to A20 and subtypes found by Olech et al. [8] were renamed from A23 to A25 and from A24 to A26; goat 2 – blue, goat 3 – black, goat 4 – yellow, goat 5 – green, goat 6 – red, goat 7 – pink.

```
                                       V4                              HV2
Consensus    TMWGIYKNCTGCANATLDRTGNGTLGTVKNINCSLPHXNESNKWTCAARQNGGKRDSLYIAG-RDFWGRVKAQYS
Cork  B1     .........S..E.....N..E....G.A.K......K.........P..RD..T.......GKK..T.I...F.
1217  B2     .....L...S..E........A.N......K......I...DQ....P.WAK.IS.......GKQ..T.....F.
K1514 A1     ...N..Q..SK.N.SS............NDLK.....R...........RK.SR........-.........K..
85/34 A2     ..M..LR..S..K........E.A..N...E..T...K....L....P.RRKRIT.......G.Q..T.......
697 A2/A3    E.MK......E.N.T.FE.I.Y......E........K....T...S..KK.R-........-............
6221  A4     ..Q...G..SQ.V....N..........S........R.K..Q......TA.K.........-............
5994  A5     ...EH.R...N.T.S......E........P......K....E...Q..RSKN....V....-.N..........
4819 A12     ......Q.....G....G...D.A....A.LS.....K...Q.......T..TR......SA-GK..E.I.....
0016 A13     ...ER.Q.......H........I..N.G........K....Q...Q..AR.NQ...V....-.N..........
8344 A17     ...EY....S..Q.E......E........P......I....T...S..TR.E.........-............
1561 A17     ...K..R..S..N.E..E...G........L......I....T...S..EGQ..........-.H..E.......
It38 A19     .....F...S..E.E......D....N...E......R.........P.WGK.GS.......G.Q..T.......
It009 A20    ...KH.Q..SN.G.......EV......N........I..TK.......A.DKR........-.N..........
4315  A25    ...RH.M..S..H....K...I......G........K....L...Q..S.KD....V....-.N..........
3201  A26    ...ER.E.....E.Q..A...E.A....A.K......R....Q......TSKTRT.......-............
Goat4 L      ....T.......C.V....E.T........V......I..AKR.......KEER........-............
Goat4 MG     ...DT.........V....E.T........V......I..AK........KEER........-............
Goat4 B      ....T.........V....E.T........V......I..AK........KEE.........-............
Goat4 SM     ...ST.T.......M....K........E........V...KR.......DET.........-............
Goat2 SM     ....T.........R....E.T........V......L...KL......KKKE.........-............
Goat2 PBMC   ....T.........V....E.T........V......L...KL......K.K..........-............
Goat7 SM     ....T.........M....E.T.......EV......I...KQ.......KD..........-..........F.
Goat6 L      ...SK.......T.M....E.........DV......V-..KL.K......EKR........-............
Goat6 MG     ...SK....K....M....E.T.......DV......V...GL......K.EKR........-K...........
Goat6 SM     ...SK.......T.M..N.E.S......E........V...KR.......DET.........-............
Goat5 SM     ...EY.......E....E..........A........L...........K..EI........-............
Goat5 PBMC   ...ST.......E.RD.G..........A........V...........K..ER........-..........F.
Goat5 B      ...ST.......G....E..........A.....V..V...KQ......EG..R........-............
Goat5 MG     ...ST.......E....E..........AD.......V...KQ......ER..R........-..........F.
Goat5 CHP    ...ST.R.....R....E.S........A..K.....V...KQ......EG...........-............

                                                      Epitope SU5
Consensus    CESNIGGLDGMMHQQXLLQRYQVIRVRAYTYGVVEMPESYXKSQVKRKR--DLKQGRKRK
Cork   B1    ......Q....L...I...K....K........I....N.A.TRIINRK--KRELSH.--
1217   B2    ......EI...L...I.................ID..TH.G-LRNGNRK--KRELSH.--
K1514  A1    ....L....S.....M.....................Q..MEA.GENR.---SRRNLQ--
85/34  A2    ...............I...K....K............K..ME..KRK..--STELQL.--
697  A2/A3   ....L..........I..................D..Q..IE..E.N..---S.RSLQ--
6221   A4    ....L..........I.................ID..Q..MQN.E.N..---A.R.LQ--
5994   A5    ....L......V...I........K.........D.....I.T.-.GRKR-STGITLR--
4819   A12   ....L..........I........K.........D..K..ME..RRK..--STELQLR--
0016   A13   ...............M........K............R..IEK.Q...QR-STELDL.--
8344   A17   ...............V..................D..K..MET.-R..KG-STEPQLR--
1561   A17   ...............V..................D..K..LDTH-R...--SPARHLE--
It38   A19   ....V.E....L...M...K....K............DQ.L.TNKR...--STEVILR--
It009  A20   ....L..........V........K.........D..N..ME--KN..K---Q.RSLQ--
4315   A25   ....L..........M........K............R..MET.-...KR-STELEL.--
3201   A26   ...............L......I.K............Q..IEK.KRKR.--STELIH.--
Goat4 L      ...............V........................G..K.....--.......--
Goat4 MG     ...............V........................G..KE....--.......--
Goat4 B      ...............V........................G.RN.....--.......—
Goat4 SM     ...............M..................Q.....V..T.....--.I..EKR..
Goat2 SM     ...............L........................G..N.....--.......--
Goat2 PBMC   ...............L........................G..N.....--.......--
Goat7 SM     ...............L........................V.NK.T...--...R...--
Goat6 L      ...............M........................V.NN.....--.......--
Goat6 MG     ...............M........................G..N.....--.......—
Goat6 SM     ...............M..................Q.....V..T.....--.I..EKR..
Goat5 SM     ...............L........................A..N.....--.......--
Goat5 PBMC   ...............L........................V.XN.....--.......—
Goat5 B      ...............V........................A.NN.....--.......--
Goat5 MG     ...............L........................V.NN.....--.......--
Goat5 CHP    ...............V................I.D.....V.T.KR.Q.SAEITLK..--
```

Figure S2. Alignment of deduced amino acid sequences of immunodominant epitope of ENV protein and variable region V4 of the SRLVs obtained in this study and reference strains representing different subtypes. Deletions are indicated by a dash (-) and identical residues are indicated by dots (.). PBMC- peripheral blood mononuclear cells, L-lung, SM-synovial membrane, B-brain, CHP-choroid plexus, MG-mammary gland.

References

8 Olech, M.; Kuźmak, J. Molecular Characterization of Small Ruminant Lentiviruses in Polish Mixed Flocks Supports Evidence of Cross Species Transmission, Dual Infection, a Recombination Event, and Reveals the Existence of New Subtypes within Group A. *Viruses* **2021**, *13*, 2529.

23 Colitti, B.; Coradduzza, E.; Puggioni, G.; Capucchio, M.T.; Reina, R.; Bertolotti, L.; Rosati, S. A new approach for Small Ruminant Lentivirus full genome characterization revealed the circulation of divergent strains. *PLoS ONE* **2019**, *14*, e0212585.
